# Bis[μ-1,2-bis­(1*H*-imidazol-1-ylmeth­yl)benzene-κ^2^ *N* ^3^:*N* ^3′^]disilver(I) bis­(4-carb­oxy­naphthalene-1-carboxyl­ate) tetra­hydrate

**DOI:** 10.1107/S1600536811018691

**Published:** 2011-05-25

**Authors:** Yan Yang, Guohui Yuan

**Affiliations:** a Department of Chemistry, Tonghua Normal University, Tonghua 134001, People’s Republic of China; b School of Chemical Engineering and Technology, Harbin Institute of Technology, Harbin 150001, People’s Republic of China

## Abstract

In the title compound, [Ag_2_(C_14_H_14_N_4_)_2_](C_12_H_7_O_4_)_2_·4H_2_O, the dinuclear dication has crystallographically imposed inversion symmetry. Each Ag^I^ ion is bicoordinated in a slightly distorted linear coordination geometry by the N atoms of two ligands, resulting in the formation of a 22-membered metallamacrocycle. In the dication, π–π inter­actions are observed between the imidazole rings, with centroid–centroid distances of 3.528 (3) Å and dihedral angles of 9.92 (9)°. The crystal structure is stabilized by inter­molecular O—H⋯O hydrogen bonds and π–π inter­actions involving the benzene rings of adjacent dications, with centroid–centroid distances of 3.651 (2) Å.

## Related literature

For the synthesis and structures of related compounds, see: Tan *et al.* (2004 ▶); Liu *et al.* (2007 ▶); Liu, Ma *et al.* (2008 ▶); Liu, Chi & Wang (2008 ▶); Sun *et al.* (2009 ▶).
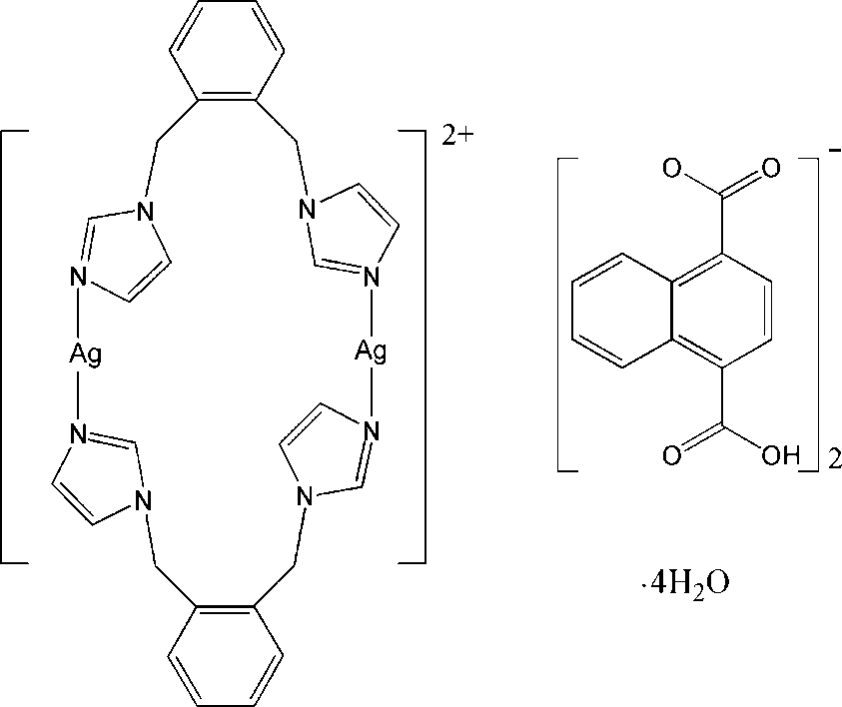


## Experimental

### 

#### Crystal data

[Ag_2_(C_14_H_14_N_4_)_2_](C_12_H_7_O_4_)_2_·4H_2_O
*M* *_r_* = 1194.74
Triclinic, 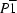

*a* = 9.6644 (5) Å
*b* = 11.3769 (12) Å
*c* = 11.8255 (5) Å
α = 109.376 (8)°
β = 95.783 (3)°
γ = 94.442 (4)°
*V* = 1211.79 (15) Å^3^
*Z* = 1
Mo *K*α radiation
μ = 0.88 mm^−1^
*T* = 293 K
0.15 × 0.12 × 0.11 mm

#### Data collection

Bruker APEX diffractometer
Absorption correction: multi-scan (*SADABS*; Bruker, 1999 ▶) *T*_min_ = 0.35, *T*_max_ = 0.59
8572 measured reflections
4904 independent reflections
3384 reflections with *I* > 2σ(*I*)
*R*_int_ = 0.023

#### Refinement

*R*[*F* ^2^ > 2σ(*F* ^2^)] = 0.029
*wR*(*F* ^2^) = 0.069
*S* = 0.89
4904 reflections
346 parameters
6 restraints
H atoms treated by a mixture of independent and constrained refinement
Δρ_max_ = 0.30 e Å^−3^
Δρ_min_ = −0.43 e Å^−3^

### 

Data collection: *SMART* (Bruker, 1997 ▶); cell refinement: *SAINT* (Bruker, 1999 ▶); data reduction: *SAINT*; program(s) used to solve structure: *SHELXS97* (Sheldrick, 2008 ▶); program(s) used to refine structure: *SHELXL97* (Sheldrick, 2008 ▶); molecular graphics: *SHELXTL* (Sheldrick, 2008 ▶); software used to prepare material for publication: *SHELXL97*.

## Supplementary Material

Crystal structure: contains datablocks global, I. DOI: 10.1107/S1600536811018691/rz2589sup1.cif

Structure factors: contains datablocks I. DOI: 10.1107/S1600536811018691/rz2589Isup2.hkl

Additional supplementary materials:  crystallographic information; 3D view; checkCIF report

## Figures and Tables

**Table 1:** Hydrogen-bond geometry (Å, °)

*D*—H⋯*A*	*D*—H	H⋯*A*	*D*⋯*A*	*D*—H⋯*A*
O2—H2*A*⋯O3^i^	0.82	1.69	2.496 (2)	166
O1*W*—H*W*11⋯O4	0.87 (2)	1.96 (2)	2.814 (3)	166 (3)
O1*W*—H*W*12⋯O2*W*^ii^	0.83 (2)	2.12 (2)	2.902 (3)	158 (3)
O2*W*—H*W*21⋯O1	0.84 (2)	1.99 (2)	2.810 (3)	164 (4)
O2*W*—H*W*22⋯O3^i^	0.88 (2)	2.13 (3)	2.841 (3)	138 (3)

Symmetry codes: (i) 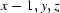; (ii) 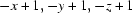.

**Fig. 1.**
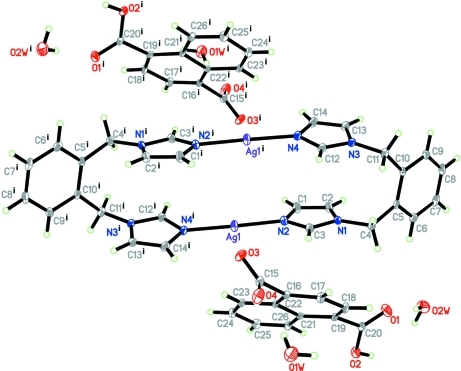
The structure of the title compound, showing displacement ellipsoids drawn at the 30% probability level. Symmetry code: (i) 2-x, -y, -z.

## supplementary crystallographic information

### Comment

The design and synthesis of silver(I) complexes have attracted intense interests of chemists (Liu, Chi & Wang, 2008; Tan *et al.*, 2004) because of the versatility of their coordination geometry (Sun *et al.*, 2009). So far, some complexes, modified by secondary nitrogen-based ligands, have been reported (Liu *et al.*, 2007). In this work, the combination of 1,2-bis(1*H*-imidazol-1-ylmethyl)benzene (1,2-bix) with naphthalene-1,4-dicarboxylic acid (1,4-H_2_ndc) and silver(I) ions resulted in the title compound, whose synthesis and structure are reported herein.

The contents of the asymmetric unit of the title compound is shown in Fig. 1. The complex, which has crystallographically imposed inversion symmetry, shows a binuclear structure, where each of silver(I) atom has a slightly distorted linear geometry and is coordinated by the N atoms from two 1,2-bix ligands. The Ag-N bond distances are within the normal range and are comparable to those observed in related N-containing compounds (Liu, Ma *et al.*, 2008). Notably, the 1,4-Hndc anion does not coordinate to the metal and acts as a counter-anion. In the dication, π-π interactions are observed between the imidazole rings (N1/N2/C1–C3 and N3/N4/C12–C14), with centroid-centroid distance of 3.528 (3) Å and dihedral angles of 9.92 (9)°. The crystal structure is stabilized by a three-dimensional network of intermolecular O—H···O hydrogen bonds (Table 1) and π-π interactions involving the benzene rings of adjacent dications, with centroid-to-centroid distances *Cg*1···*Cg*1^i^ =  3.651 (2) Å [*Cg*1 is the centroid of the C5–C10 ring; symmetry code: (i) 1-x, -y, 1-z].

### Experimental

A mixture of AgNO_3_^.^2H_2_O (0.5 mmol), naphthalene-1,4-dicarboxylic acid (0.5 mmol), 1,2-bis(1*H*-imidazol-1-ylmethyl)benzene (0.5 mmol) in H_2_O (12 ml) was adjusted to pH = 5-6 by addition of aqueous NaOH solution, and heated at 145°C for 2 days. After the mixture was slowly cooled to room temperature, crystals of the title compound suitable for X-ray analysis were obtained (yield 33%).

### Refinement

Water hydrogen atoms were located in difference Fourier maps and refined isotropically, with distance restraints of O—H = 0.85 (1) and H···H = 1.35 (1) Å and with U_iso_(H) = 1.5U_eq_(O). All other H atoms were positioned geometrically (C—H = 0.93 Å, O—H = 0.82 Å) and refined as riding, with *U*_iso_(H)=1.2*U*_eq_(C, O).

### Crystal data

**Table:**

[Ag_2_(C_14_H_14_N_4_)_2_](C_12_H_7_O_4_)_2_·4H_2_O	*Z* = 1
*M**_r_* = 1194.74	*F*(000) = 608
Triclinic, *P*1	*D*_x_ = 1.637 Mg m^−^^3^
Hall symbol: -P 1	Mo *K*α radiation, λ = 0.71073 Å
*a* = 9.6644 (5) Å	Cell parameters from 4904 reflections
*b* = 11.3769 (12) Å	θ = 1.8–26.4°
*c* = 11.8255 (5) Å	µ = 0.88 mm^−^^1^
α = 109.376 (8)°	*T* = 293 K
β = 95.783 (3)°	Block, pale yellow
γ = 94.442 (4)°	0.15 × 0.12 × 0.11 mm
*V* = 1211.79 (15) Å^3^	

### Data collection

**Table:**

Bruker APEX diffractometer	4904 independent reflections
Radiation source: fine-focus sealed tube	3384 reflections with *I* > 2σ(*I*)
graphite	*R*_int_ = 0.023
φ and ω scans	θ_max_ = 26.4°, θ_min_ = 1.8°
Absorption correction: multi-scan (*SADABS*; Bruker, 1999)	*h* = −11→12
*T*_min_ = 0.35, *T*_max_ = 0.59	*k* = −14→12
8572 measured reflections	*l* = −14→11

### Refinement

**Table:**

Refinement on *F*^2^	Primary atom site location: structure-invariant direct methods
Least-squares matrix: full	Secondary atom site location: difference Fourier map
*R*[*F*^2^ > 2σ(*F*^2^)] = 0.029	Hydrogen site location: inferred from neighbouring sites
*wR*(*F*^2^) = 0.069	H atoms treated by a mixture of independent and constrained refinement
*S* = 0.89	*w* = 1/[σ^2^(*F*_o_^2^) + (0.0385*P*)^2^] where *P* = (*F*_o_^2^ + 2*F*_c_^2^)/3
4904 reflections	(Δ/σ)_max_ = 0.001
346 parameters	Δρ_max_ = 0.30 e Å^−^^3^
6 restraints	Δρ_min_ = −0.43 e Å^−^^3^

### Special details

**Table:**

Geometry. All esds (except the esd in the dihedral angle between two l.s. planes) are estimated using the full covariance matrix. The cell esds are taken into account individually in the estimation of esds in distances, angles and torsion angles; correlations between esds in cell parameters are only used when they are defined by crystal symmetry. An approximate (isotropic) treatment of cell esds is used for estimating esds involving l.s. planes.
Refinement. Refinement of F^2^ against ALL reflections. The weighted R-factor wR and goodness of fit S are based on F^2^, conventional R-factors R are based on F, with F set to zero for negative F^2^. The threshold expression of F^2^ > 2sigma(F^2^) is used only for calculating R-factors(gt) etc. and is not relevant to the choice of reflections for refinement. R-factors based on F^2^ are statistically about twice as large as those based on F, and R- factors based on ALL data will be even larger.

### Fractional atomic coordinates and isotropic or equivalent isotropic displacement parameters (Å^2^)

**Table:**

	*x*	*y*	*z*	*U*_iso_*/*U*_eq_	
C1	0.6791 (3)	−0.0550 (2)	0.0091 (2)	0.0501 (6)	
H1	0.6710	−0.1140	−0.0684	0.060*	
C2	0.6045 (3)	−0.0634 (2)	0.0974 (2)	0.0458 (6)	
H2	0.5361	−0.1279	0.0919	0.055*	
C3	0.7469 (2)	0.1091 (2)	0.1643 (2)	0.0424 (6)	
H3	0.7937	0.1857	0.2157	0.051*	
C4	0.5997 (3)	0.0755 (2)	0.3151 (2)	0.0442 (6)	
H4A	0.5347	0.1374	0.3205	0.053*	
H4B	0.6790	0.1137	0.3774	0.053*	
C5	0.5284 (2)	−0.0355 (2)	0.33815 (18)	0.0341 (5)	
C6	0.3839 (2)	−0.0568 (2)	0.3188 (2)	0.0434 (6)	
H6	0.3335	−0.0034	0.2905	0.052*	
C7	0.3121 (2)	−0.1558 (3)	0.3405 (2)	0.0479 (6)	
H7	0.2147	−0.1685	0.3266	0.058*	
C8	0.3846 (3)	−0.2340 (2)	0.3823 (2)	0.0460 (6)	
H8	0.3371	−0.3008	0.3968	0.055*	
C9	0.5289 (3)	−0.2138 (2)	0.4032 (2)	0.0420 (6)	
H9	0.5780	−0.2673	0.4324	0.050*	
C10	0.6025 (2)	−0.1153 (2)	0.38161 (18)	0.0329 (5)	
C11	0.7610 (2)	−0.0995 (2)	0.4061 (2)	0.0443 (6)	
H11A	0.7950	−0.0110	0.4437	0.053*	
H11B	0.7903	−0.1418	0.4618	0.053*	
C12	0.9213 (2)	−0.0917 (2)	0.2549 (2)	0.0402 (6)	
H12	0.9636	−0.0104	0.2967	0.048*	
C13	0.7869 (2)	−0.2677 (2)	0.2088 (2)	0.0465 (6)	
H13	0.7210	−0.3304	0.2117	0.056*	
C14	0.8654 (3)	−0.2743 (2)	0.1202 (2)	0.0510 (7)	
H14	0.8624	−0.3432	0.0500	0.061*	
C15	1.0338 (2)	0.4758 (2)	0.2815 (2)	0.0376 (5)	
C16	0.87617 (19)	0.45208 (19)	0.27283 (19)	0.0296 (5)	
C17	0.8186 (2)	0.4650 (2)	0.3765 (2)	0.0392 (6)	
H17	0.8761	0.4916	0.4503	0.047*	
C18	0.6738 (2)	0.4386 (2)	0.3729 (2)	0.0385 (5)	
H18	0.6374	0.4426	0.4438	0.046*	
C19	0.58560 (19)	0.40728 (19)	0.26764 (19)	0.0294 (5)	
C20	0.4319 (2)	0.3805 (2)	0.2734 (2)	0.0353 (5)	
C21	0.64007 (19)	0.39844 (18)	0.15716 (18)	0.0255 (4)	
C22	0.78814 (19)	0.41758 (18)	0.16013 (18)	0.0246 (4)	
C23	0.8428 (2)	0.40779 (19)	0.05138 (19)	0.0314 (5)	
H23	0.9393	0.4196	0.0527	0.038*	
C24	0.7584 (2)	0.3816 (2)	−0.0548 (2)	0.0386 (5)	
H24	0.7967	0.3765	−0.1251	0.046*	
C25	0.6131 (2)	0.3624 (2)	−0.0577 (2)	0.0417 (6)	
H25	0.5551	0.3439	−0.1306	0.050*	
C26	0.5555 (2)	0.3705 (2)	0.04427 (19)	0.0357 (5)	
H26	0.4587	0.3575	0.0400	0.043*	
O1	0.39354 (17)	0.3166 (2)	0.3301 (2)	0.0755 (7)	
O2	0.35024 (15)	0.43133 (18)	0.21712 (17)	0.0592 (5)	
H2A	0.2689	0.4124	0.2246	0.089*	
O1W	1.0555 (2)	0.7995 (2)	0.5309 (2)	0.0790 (6)	
HW11	1.053 (4)	0.725 (2)	0.477 (3)	0.119*	
HW12	0.991 (3)	0.788 (3)	0.569 (3)	0.119*	
O3	1.09219 (15)	0.38750 (17)	0.21193 (16)	0.0525 (4)	
O2W	0.1287 (2)	0.1769 (2)	0.2855 (3)	0.0908 (7)	
HW21	0.204 (3)	0.224 (3)	0.313 (4)	0.136*	
HW22	0.086 (4)	0.210 (4)	0.237 (3)	0.136*	
O4	1.09571 (17)	0.57350 (19)	0.35306 (17)	0.0630 (5)	
Ag1	0.91046 (2)	0.11314 (2)	−0.04497 (2)	0.05935 (10)	
N1	0.64894 (18)	0.04051 (16)	0.19530 (16)	0.0346 (4)	
N2	0.7687 (2)	0.05428 (19)	0.05184 (18)	0.0461 (5)	
N3	0.82247 (17)	−0.15126 (17)	0.29391 (16)	0.0350 (4)	
N4	0.95103 (19)	−0.16342 (18)	0.14922 (18)	0.0443 (5)	

### Atomic displacement parameters (Å^2^)

**Table:**

	*U*^11^	*U*^22^	*U*^33^	*U*^12^	*U*^13^	*U*^23^
C1	0.0624 (17)	0.0469 (16)	0.0398 (14)	0.0010 (14)	0.0177 (12)	0.0115 (12)
C2	0.0520 (15)	0.0433 (15)	0.0415 (14)	−0.0066 (12)	0.0128 (11)	0.0145 (12)
C3	0.0452 (14)	0.0336 (13)	0.0498 (16)	−0.0008 (11)	0.0144 (12)	0.0151 (12)
C4	0.0566 (15)	0.0378 (14)	0.0404 (14)	0.0063 (12)	0.0215 (12)	0.0118 (11)
C5	0.0379 (13)	0.0377 (13)	0.0275 (12)	0.0048 (10)	0.0158 (10)	0.0089 (10)
C6	0.0396 (13)	0.0542 (16)	0.0362 (13)	0.0137 (12)	0.0109 (11)	0.0118 (12)
C7	0.0315 (13)	0.0632 (18)	0.0379 (14)	−0.0060 (12)	0.0124 (11)	0.0029 (13)
C8	0.0487 (15)	0.0441 (15)	0.0401 (14)	−0.0110 (13)	0.0201 (12)	0.0073 (12)
C9	0.0525 (15)	0.0419 (14)	0.0365 (13)	0.0076 (12)	0.0177 (11)	0.0162 (11)
C10	0.0337 (12)	0.0372 (13)	0.0272 (11)	0.0032 (10)	0.0134 (9)	0.0080 (10)
C11	0.0362 (13)	0.0569 (16)	0.0398 (14)	0.0055 (12)	0.0122 (11)	0.0143 (12)
C12	0.0309 (12)	0.0368 (13)	0.0545 (16)	0.0030 (10)	0.0133 (11)	0.0156 (12)
C13	0.0425 (14)	0.0290 (13)	0.0708 (18)	0.0033 (11)	0.0266 (13)	0.0157 (13)
C14	0.0514 (15)	0.0351 (14)	0.0646 (17)	0.0075 (12)	0.0272 (13)	0.0084 (13)
C15	0.0227 (11)	0.0521 (15)	0.0397 (14)	−0.0015 (11)	0.0024 (10)	0.0198 (12)
C16	0.0181 (10)	0.0340 (12)	0.0381 (13)	0.0033 (9)	0.0047 (9)	0.0138 (10)
C17	0.0228 (11)	0.0583 (16)	0.0344 (13)	0.0021 (11)	−0.0020 (9)	0.0155 (12)
C18	0.0269 (11)	0.0567 (15)	0.0342 (13)	0.0049 (11)	0.0103 (10)	0.0166 (11)
C19	0.0186 (10)	0.0339 (12)	0.0369 (13)	0.0056 (9)	0.0070 (9)	0.0122 (10)
C20	0.0224 (11)	0.0426 (14)	0.0408 (13)	0.0032 (10)	0.0089 (10)	0.0131 (11)
C21	0.0194 (10)	0.0240 (11)	0.0330 (12)	0.0043 (8)	0.0046 (9)	0.0090 (9)
C22	0.0191 (10)	0.0214 (10)	0.0321 (12)	0.0019 (8)	0.0042 (8)	0.0076 (9)
C23	0.0212 (10)	0.0320 (12)	0.0413 (13)	0.0025 (9)	0.0105 (10)	0.0114 (10)
C24	0.0377 (13)	0.0426 (14)	0.0344 (13)	0.0022 (11)	0.0095 (10)	0.0109 (11)
C25	0.0385 (13)	0.0503 (15)	0.0303 (13)	0.0017 (11)	−0.0035 (10)	0.0092 (11)
C26	0.0216 (11)	0.0423 (14)	0.0393 (13)	0.0015 (10)	0.0028 (10)	0.0095 (11)
O1	0.0282 (9)	0.1157 (17)	0.1216 (18)	0.0090 (10)	0.0207 (10)	0.0889 (15)
O2	0.0200 (8)	0.0928 (14)	0.0906 (14)	0.0163 (9)	0.0166 (9)	0.0605 (12)
O1W	0.0747 (15)	0.0615 (14)	0.0900 (17)	−0.0045 (12)	0.0228 (12)	0.0110 (12)
O3	0.0211 (8)	0.0636 (12)	0.0697 (12)	0.0109 (8)	0.0091 (8)	0.0167 (10)
O2W	0.0619 (14)	0.0779 (17)	0.146 (2)	−0.0041 (12)	0.0282 (14)	0.0553 (16)
O4	0.0303 (9)	0.0721 (13)	0.0657 (12)	−0.0156 (9)	0.0044 (9)	0.0014 (11)
Ag1	0.05254 (14)	0.07269 (17)	0.07007 (17)	0.00896 (11)	0.03247 (11)	0.03988 (13)
N1	0.0398 (11)	0.0306 (10)	0.0364 (11)	0.0028 (9)	0.0122 (8)	0.0139 (9)
N2	0.0498 (12)	0.0460 (13)	0.0540 (14)	0.0089 (10)	0.0240 (10)	0.0268 (11)
N3	0.0284 (10)	0.0357 (11)	0.0442 (11)	0.0057 (8)	0.0121 (8)	0.0155 (9)
N4	0.0412 (11)	0.0414 (12)	0.0590 (14)	0.0097 (10)	0.0258 (10)	0.0221 (10)

### Geometric parameters (Å, °)

**Table:**

C1—C2	1.351 (3)	C14—H14	0.9300
C1—N2	1.371 (3)	C15—O4	1.217 (3)
C1—H1	0.9300	C15—O3	1.279 (3)
C2—N1	1.355 (3)	C15—C16	1.513 (3)
C2—H2	0.9300	C16—C17	1.365 (3)
C3—N2	1.315 (3)	C16—C22	1.424 (3)
C3—N1	1.336 (3)	C17—C18	1.402 (3)
C3—H3	0.9300	C17—H17	0.9300
C4—N1	1.477 (3)	C18—C19	1.360 (3)
C4—C5	1.508 (3)	C18—H18	0.9300
C4—H4A	0.9700	C19—C21	1.433 (3)
C4—H4B	0.9700	C19—C20	1.506 (3)
C5—C6	1.381 (3)	C20—O1	1.203 (3)
C5—C10	1.393 (3)	C20—O2	1.273 (3)
C6—C7	1.387 (3)	C21—C26	1.417 (3)
C6—H6	0.9300	C21—C22	1.426 (3)
C7—C8	1.359 (4)	C22—C23	1.413 (3)
C7—H7	0.9300	C23—C24	1.356 (3)
C8—C9	1.379 (3)	C23—H23	0.9300
C8—H8	0.9300	C24—C25	1.400 (3)
C9—C10	1.389 (3)	C24—H24	0.9300
C9—H9	0.9300	C25—C26	1.357 (3)
C10—C11	1.515 (3)	C25—H25	0.9300
C11—N3	1.468 (3)	C26—H26	0.9300
C11—H11A	0.9700	O2—H2A	0.8200
C11—H11B	0.9700	O1W—HW11	0.874 (17)
C12—N4	1.320 (3)	O1W—HW12	0.833 (17)
C12—N3	1.334 (3)	O2W—HW21	0.838 (18)
C12—H12	0.9300	O2W—HW22	0.882 (18)
C13—C14	1.340 (3)	Ag1—N2	2.0783 (17)
C13—N3	1.364 (3)	Ag1—N4^i^	2.0787 (17)
C13—H13	0.9300	N4—Ag1^i^	2.0787 (17)
C14—N4	1.373 (3)		
			
C2—C1—N2	109.2 (2)	O3—C15—C16	115.7 (2)
C2—C1—H1	125.4	C17—C16—C22	119.93 (18)
N2—C1—H1	125.4	C17—C16—C15	118.49 (18)
C1—C2—N1	106.6 (2)	C22—C16—C15	121.58 (17)
C1—C2—H2	126.7	C16—C17—C18	120.8 (2)
N1—C2—H2	126.7	C16—C17—H17	119.6
N2—C3—N1	111.2 (2)	C18—C17—H17	119.6
N2—C3—H3	124.4	C19—C18—C17	121.12 (19)
N1—C3—H3	124.4	C19—C18—H18	119.4
N1—C4—C5	112.57 (18)	C17—C18—H18	119.4
N1—C4—H4A	109.1	C18—C19—C21	120.19 (17)
C5—C4—H4A	109.1	C18—C19—C20	117.02 (18)
N1—C4—H4B	109.1	C21—C19—C20	122.78 (18)
C5—C4—H4B	109.1	O1—C20—O2	124.30 (19)
H4A—C4—H4B	107.8	O1—C20—C19	120.2 (2)
C6—C5—C10	118.62 (19)	O2—C20—C19	115.45 (18)
C6—C5—C4	118.8 (2)	C26—C21—C22	117.74 (17)
C10—C5—C4	122.5 (2)	C26—C21—C19	123.92 (17)
C5—C6—C7	121.6 (2)	C22—C21—C19	118.34 (17)
C5—C6—H6	119.2	C23—C22—C16	121.85 (17)
C7—C6—H6	119.2	C23—C22—C21	118.69 (18)
C8—C7—C6	119.7 (2)	C16—C22—C21	119.38 (17)
C8—C7—H7	120.2	C24—C23—C22	121.85 (19)
C6—C7—H7	120.2	C24—C23—H23	119.1
C7—C8—C9	119.7 (2)	C22—C23—H23	119.1
C7—C8—H8	120.2	C23—C24—C25	119.4 (2)
C9—C8—H8	120.2	C23—C24—H24	120.3
C8—C9—C10	121.4 (2)	C25—C24—H24	120.3
C8—C9—H9	119.3	C26—C25—C24	121.0 (2)
C10—C9—H9	119.3	C26—C25—H25	119.5
C9—C10—C5	119.0 (2)	C24—C25—H25	119.5
C9—C10—C11	118.4 (2)	C25—C26—C21	121.34 (19)
C5—C10—C11	122.57 (19)	C25—C26—H26	119.3
N3—C11—C10	111.23 (18)	C21—C26—H26	119.3
N3—C11—H11A	109.4	C20—O2—H2A	109.5
C10—C11—H11A	109.4	HW11—O1W—HW12	101 (2)
N3—C11—H11B	109.4	HW21—O2W—HW22	103 (2)
C10—C11—H11B	109.4	N2—Ag1—N4^i^	177.04 (8)
H11A—C11—H11B	108.0	C3—N1—C2	107.41 (18)
N4—C12—N3	111.1 (2)	C3—N1—C4	124.93 (19)
N4—C12—H12	124.5	C2—N1—C4	127.65 (18)
N3—C12—H12	124.5	C3—N2—C1	105.65 (18)
C14—C13—N3	106.6 (2)	C3—N2—Ag1	128.88 (16)
C14—C13—H13	126.7	C1—N2—Ag1	125.46 (16)
N3—C13—H13	126.7	C12—N3—C13	107.32 (19)
C13—C14—N4	109.5 (2)	C12—N3—C11	126.2 (2)
C13—C14—H14	125.3	C13—N3—C11	126.49 (18)
N4—C14—H14	125.3	C12—N4—C14	105.53 (18)
O4—C15—O3	124.9 (2)	C12—N4—Ag1^i^	126.65 (15)
O4—C15—C16	119.4 (2)	C14—N4—Ag1^i^	127.71 (16)

Symmetry codes: (i) −*x*+2, −*y*, −*z*.

### Hydrogen-bond geometry (Å, °)

**Table:**

*D*—H···*A*	*D*—H	H···*A*	*D*···*A*	*D*—H···*A*
O2—H2A···O3^ii^	0.82	1.69	2.496 (2)	166
O1W—HW11···O4	0.87 (2)	1.96 (2)	2.814 (3)	166 (3)
O1W—HW12···O2W^iii^	0.83 (2)	2.12 (2)	2.902 (3)	158 (3)
O2W—HW21···O1	0.84 (2)	1.99 (2)	2.810 (3)	164 (4)
O2W—HW22···O3^ii^	0.88 (2)	2.13 (3)	2.841 (3)	138 (3)

Symmetry codes: (ii) *x*−1, *y*, *z*; (iii) −*x*+1, −*y*+1, −*z*+1.
